# Supplementary material for: Enhancing biological signals and detection rates in single-cell RNA-seq experiments with cDNA library equalization
Source: Nucleic Acids Res. 2021 Nov 24;50(2):e12. doi: 10.1093/nar/gkab1071 (PMC8789062; doi:10.1093/nar/gkab1071)

Supplementary Methods

Simulation Parameters

The table below contains all the main simulation parameters and an indication of whether they are estimated from the data or need to be input from the user.

Parameter	Notation	Usage/Default
Protocol to simulate from (plate-based or droplet/10X)	-	Input by user
Whether to use UMI	-	Input by user
Number of cells	N	Estimated from data
Number of genes	G	Estimated from data
Gene means	μ_g	Estimated from data
Total transcripts	-	300,000
Degree of heterogeneity	ω_j	Estimated from data
Capture efficiency	λ_j	Estimated from data
Number of pre-amplification cycles	C	Input by user
Number of amplification cycles	C_2	Input by user
Pre-amplification efficiency	ρ_j	Input by user
Amplification efficiency	$\rho_{2,j}$	Input by user
Equalization amount	-	Input by user
Tagmentation efficiency	γ_j	Input by user
Total sequencing depth	R	Estimated from data

For all simulations described in the paper, we used the default distribution of Normal(0.95, 0.02) to generate amplification and tagmentation efficiencies (in Scaffold any values > 1 are truncated to 1). For all simulations of Smart-seq datasets we set $C = 18$ and $C_2 = 12$, as used in the EC and TB experiments and recommended in the C1 Fluidigm manufacturer manual. For the 10X and UMI datasets we set $C_2 = 12$. The equalization amount is the user input quantile from which q^* is estimated. For the Smart-seq experimental data, unEQ was set to 0.67 as the cells were diluted to be within a wide range of concentrations; this resulted in the dilution of only the largest concentrations thus, we considered this unequalized. For EQ experiments, the equalization amount was set to 0 to indicate full equalization. In simulations of unEQ and EQ TB and EC data, parameters were only estimated on the unEQ experimental data. All parameters were held constant to simulate the EQ data, except the equalization amount was set to 0. For the UMI and 10X datasets, the equalization amount was set to 1 to indicate no equalization. For the

Smart-seq3 datasets, the equalization was performed within a relatively wide-range of concentrations (100-200pg μl^{-1}). However, when simulating data we found setting the Fibroblast to no equalization and the HCA to 0.67 produced the best matching datasets in terms of the cell-specific and gene-specific properties (the equalization amount parameter was run for a coarse grid: 0, .33, .67, 1). This setting is in line with the unEQ EC and TB simulations in that minimal equalization was similar to no equalization. For the Smart-seq3 datasets, we used Scaffold to simulate from the read count matrix using the UMI option which produced both a read count matrix and UMI count matrix. We compared the simulated UMI count matrix to the original UMI counts. We did not simulate any data directly from the original UMI count matrix for the Smart-seq3 data in order to replicate the experimental process of generating UMI counts.

For the multiple population simulation in Figure 5, the number of cells for Population 1 was set to 50 cells and 40 cells for Population 2. The degree of heterogeneity was set to be simulated from Uniform(0.5, 2), the fold-changes were simulated from Normal(1.25, 0.4), and 15% of genes were set to have distinct expression. All other parameters were estimated from the unEQ EC dataset as a reference. The average silhouette distance was calculated from the distance matrix on the first two components of the t-SNE representation. The t-SNE components were generated using the runTSNE function from the scater v1.12.2 R package with perplexity=10, n_dimred=10, and dimred="PCA". The simulations were averaged over 25 runs.

For the continuous population simulation we simulated 100 cells with the population heterogeneity sampled from a Uniform(1,1) so that all heterogeneity was introduced from the dynamic genes. We set 20% of the genes to have dynamic expression and all other parameters were estimated from the unEQ EC dataset as a reference. On the simulated data, we used the SCORPIUS v1.0.7 R package to infer a trajectory through the cells. We used the reduce_dimensionality function with "pearson" and all other parameters default. We then fit a polynomial regression of degree two on the scaled expression and trajectory and obtained the overall model fit p-value. P-values were adjusted using FDR. Genes with an adjusted p-value < .05 were considered dynamic. We compared this to the list of simulated dynamic genes and used the pROC v1.17.0.1 R package to obtain the AUC. The simulations were averaged over 25 runs.

Simulation run-times

Average run-times for Scaffold were estimated using the unEQ TB and 10X datasets with varying number of cells to simulate. All times include both the parameter estimation and simulation; times are averaged over 10 runs.

Number of cells	Average runtime (minutes)	Protocol
100	0.14	C1, nonUMI
1000	1.55	C1, nonUMI
5000	9.41	C1, nonUMI
1000	3.73	UMI/10X
5000	24.09	UMI/10X

Supplementary Figure 1. Cell-cell Pearson correlations across experiments. Cells are in the same order along the axes for the two experiments being compared. The strong diagonal correlation verifies that the cells are indeed the same across experiments.

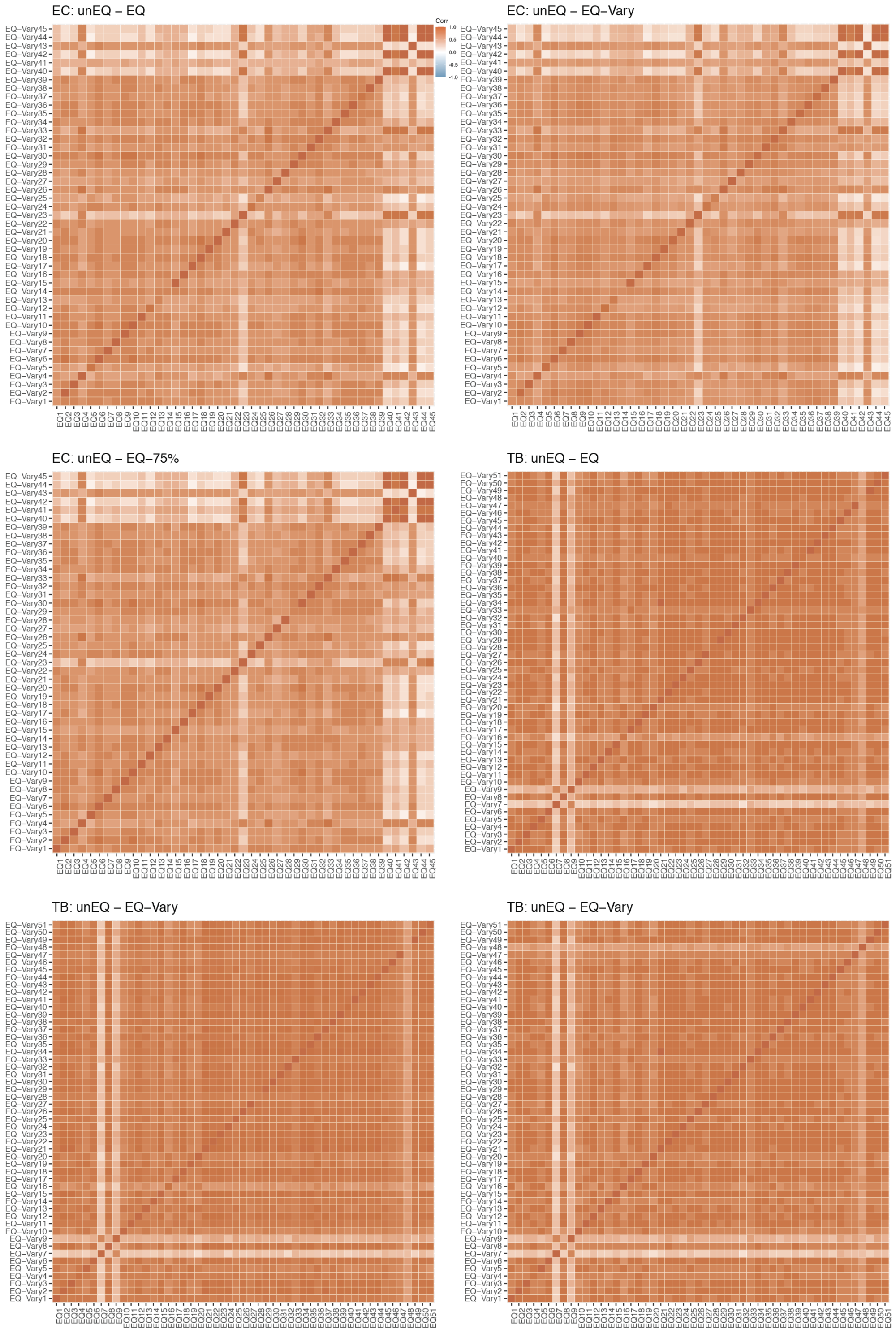

Supplementary Figure 2. Outliers cells were removed from further analysis for the TB and EC experimental data. Outliers were identified as those having log10 total counts < 5.4 or the percent of counts in the top 50 genes was > 31%.

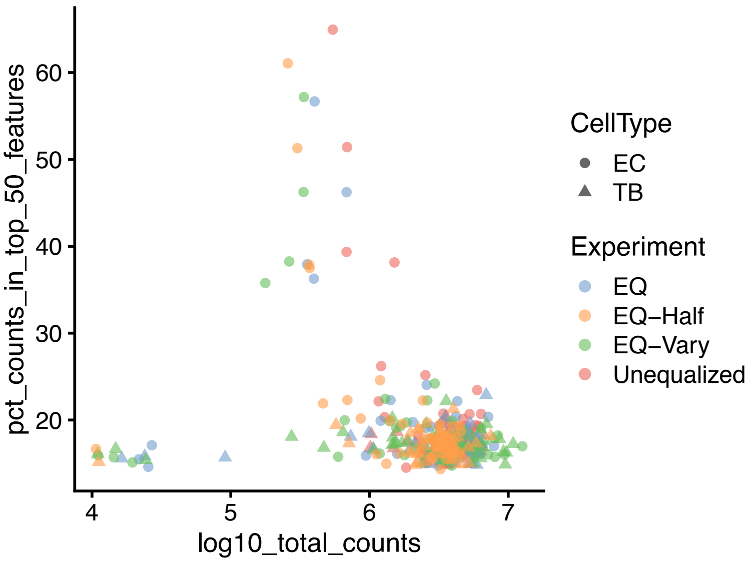

Supplementary Figure 3. A. Density plots of the distribution of estimated count-depth rates for the unEQ TB dataset with genes grouped by expression level (left) and the mode of each group's slope distribution (right). The median absolute deviation (MAD) of the slope modes from one is used to quantify the variability in the count-depth rate. B-F. Cell-specific and gene-specific properties of the data simulated based on the unEQ TB dataset. Gene-level properties are shown for a random sample of 200 genes.

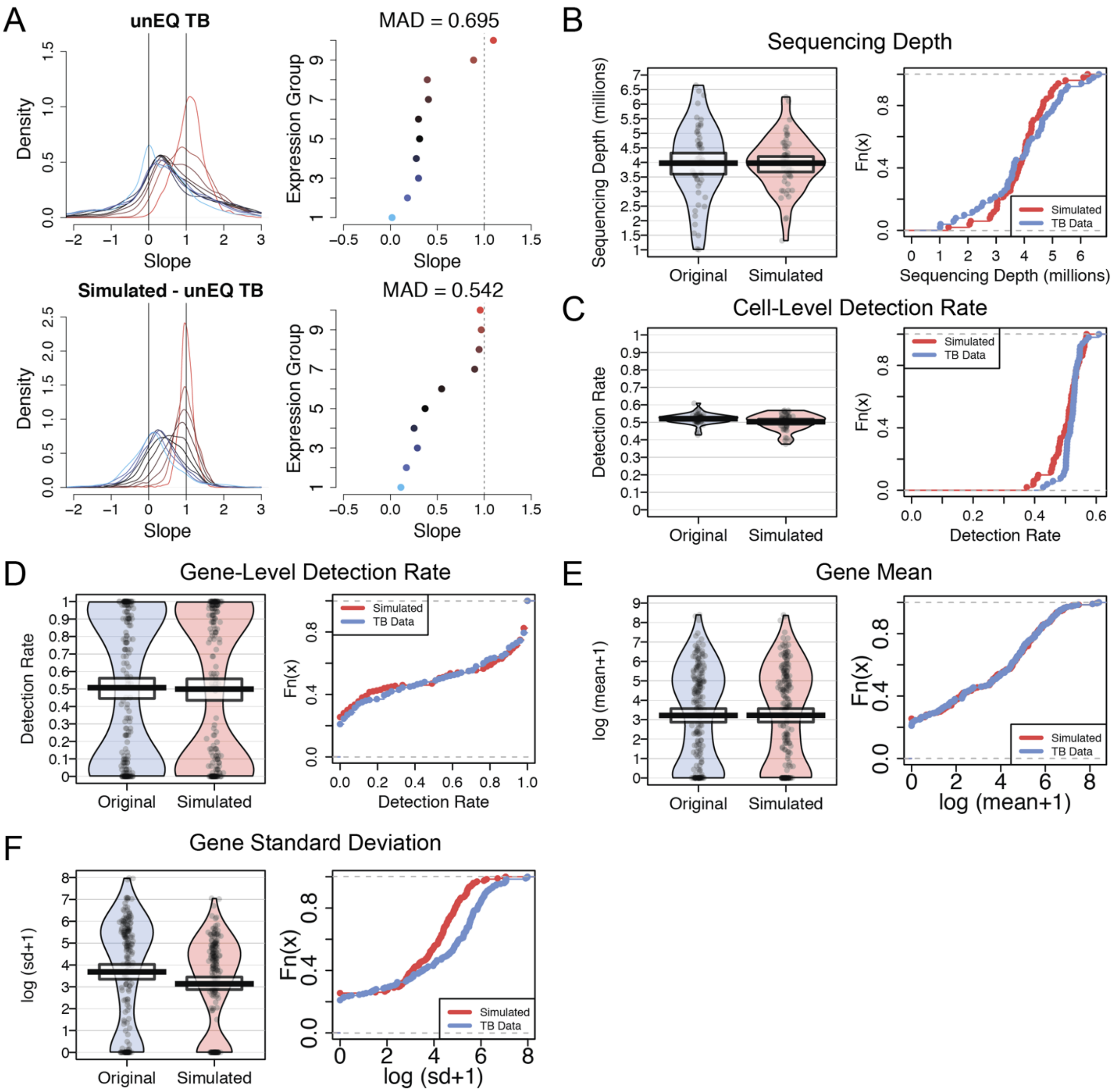

Supplementary Figure 4. A. Density plots of the distribution of estimated count-depth rates for the unEQ EC dataset with genes grouped by expression level (left) and the mode of each group's slope distribution (right). The median absolute deviation (MAD) of the slope modes from one is used to quantify the variability in the count-depth rate. B. Similar to A for one simulated dataset with parameters set to match the unEQ EC dataset. C. Same as B but the simulation was done assuming equalization of cDNA concentrations. D. The MAD is shown for 100 simulations holding all parameters constant, and varying the amount of equalization. E.) The MAD for 100 simulations holding all parameters constant, and varying the sequencing depth as X times the original, where X = 0.25, 0.5, 1, 2, 3 .

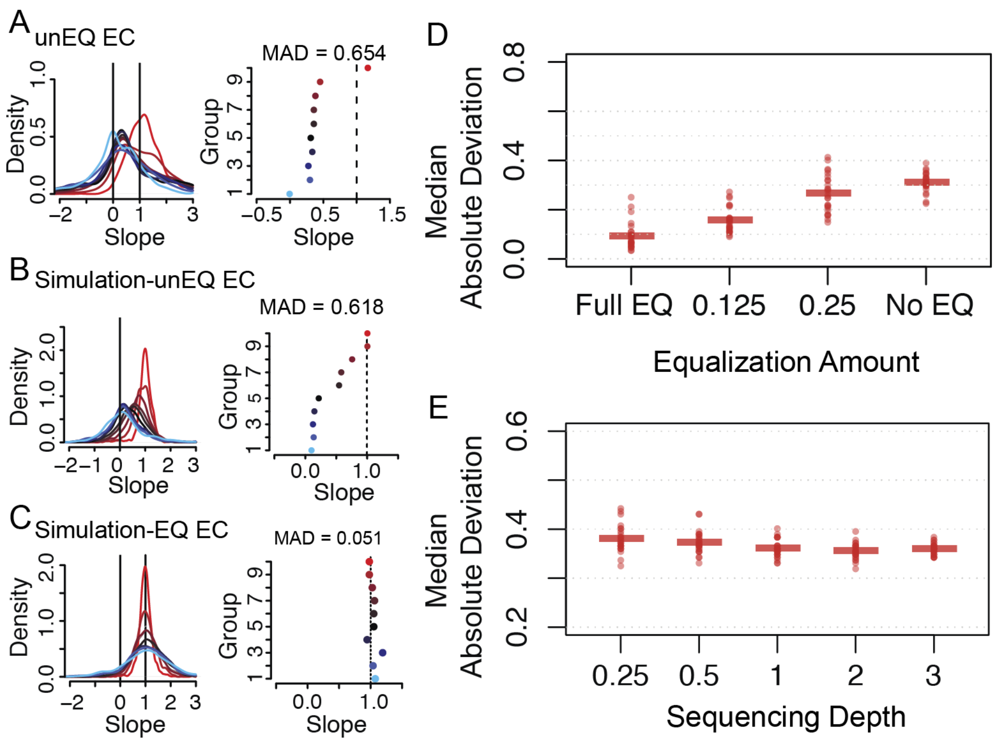

Supplementary Figure 5. Detection rate differences for a random split of cells in each of the EC unEQ (A) and TB unEQ (B) datasets. Genes were divided into four equally sized groups based on their median nonzero expression. For each gene, the difference between the detection rate in the random data splits was calculated. The cumulative distribution curve is shown for the detection rate differences for genes in each expression group. The two horizonal dotted lines indicate the proportion of genes that decrease in detection rate (bottom line) and one minus the proportion of genes that increase in detection rate (top line).

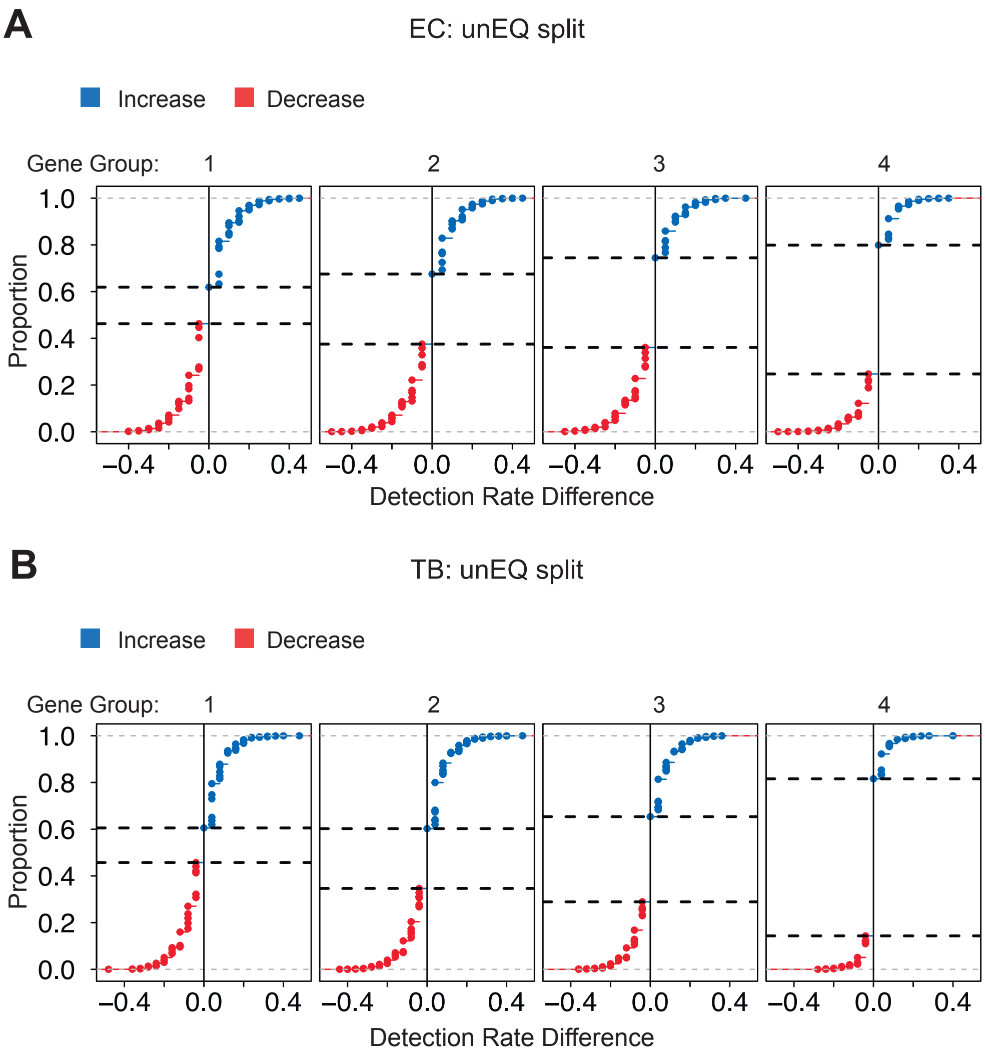

Supplementary Figure 6. Detection rate differences between the EQ and EQ-Vary datasets for the EC (A) and TB (B) conditions. Genes were divided into four equally sized groups based on their median nonzero expression. For each gene, the difference between the detection rate in the EQ versus EQ-Vary experiments was calculated. The cumulative distribution curve is shown for the detection rate differences for genes in each expression group. The two horizonal dotted lines indicate the proportion of genes that decrease in detection rate (bottom line) and one minus the proportion of genes that increase in detection rate (top line).

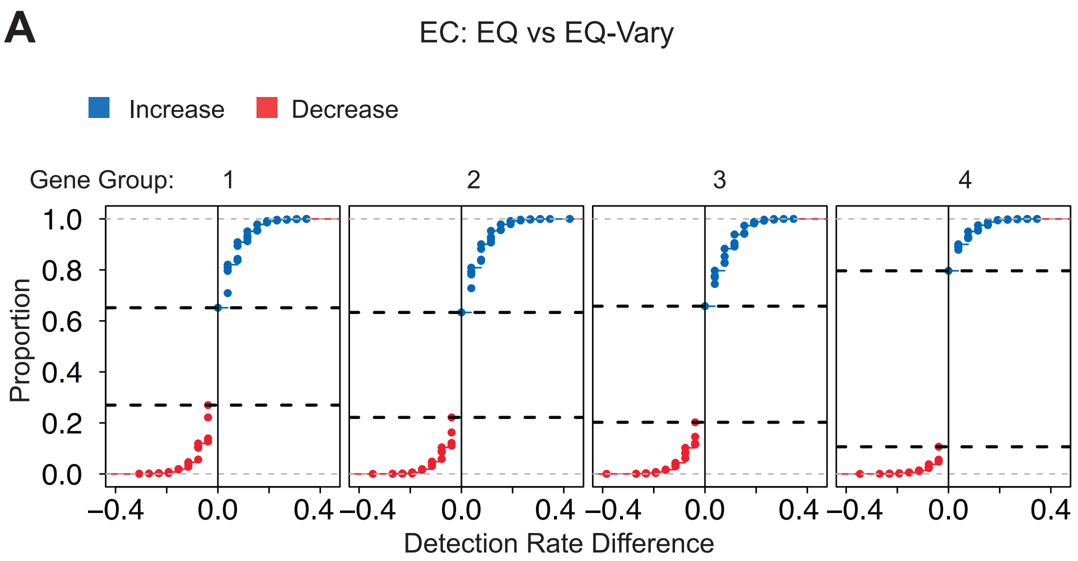

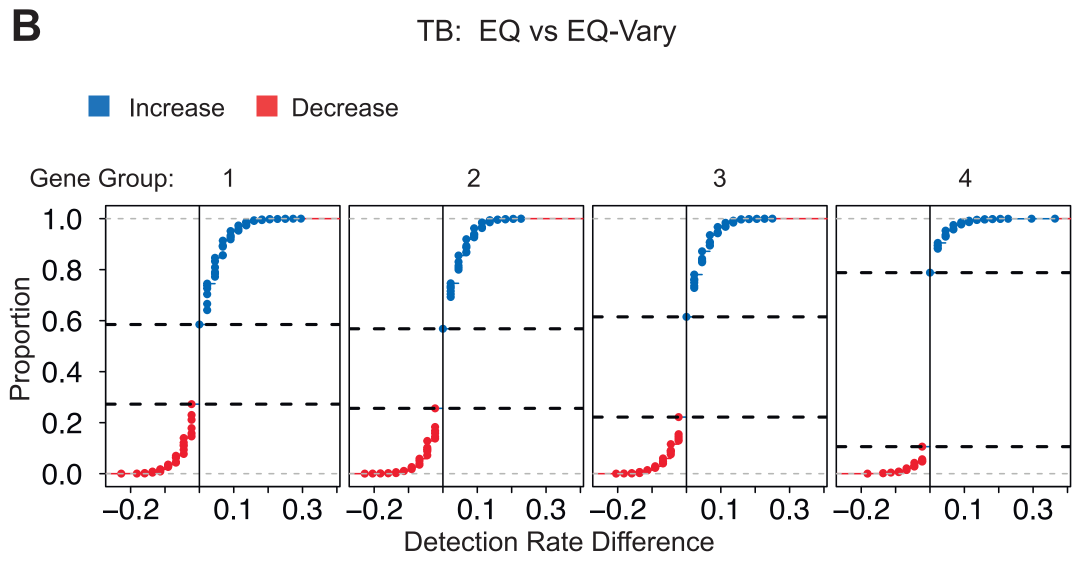

Supplementary Figure 7. The count-depth rate is estimated as a median quantile regression of log expression versus log sequencing depth. A low, moderate, and highly expressed gene are shown having a count-depth rate of 0.05, 0.53, and 0.96, respectively.

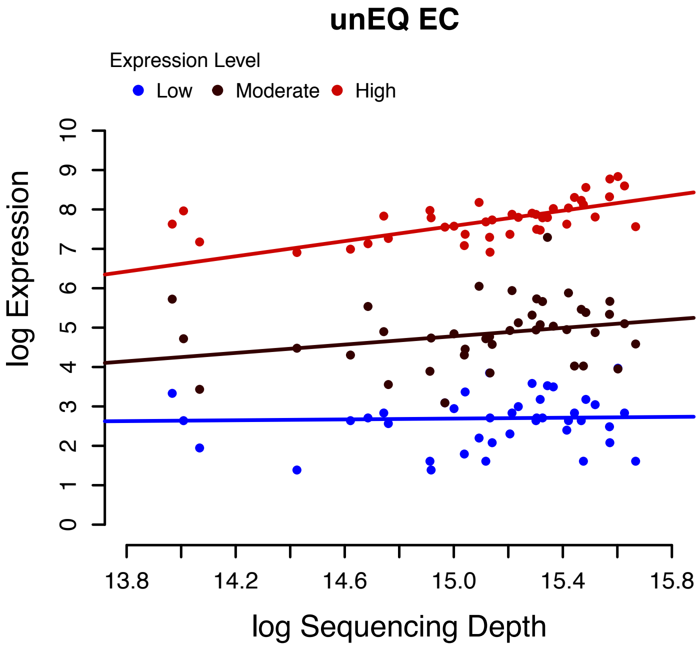

Supplementary Figure 8. Count-depth relationships for all EC and TB experiments. A. Density plots of the distribution of estimated count-depth rates for the EC datasets with genes grouped by expression level (left) and the mode of each group's slope distribution (right). B. Similar to A for the TB datasets.

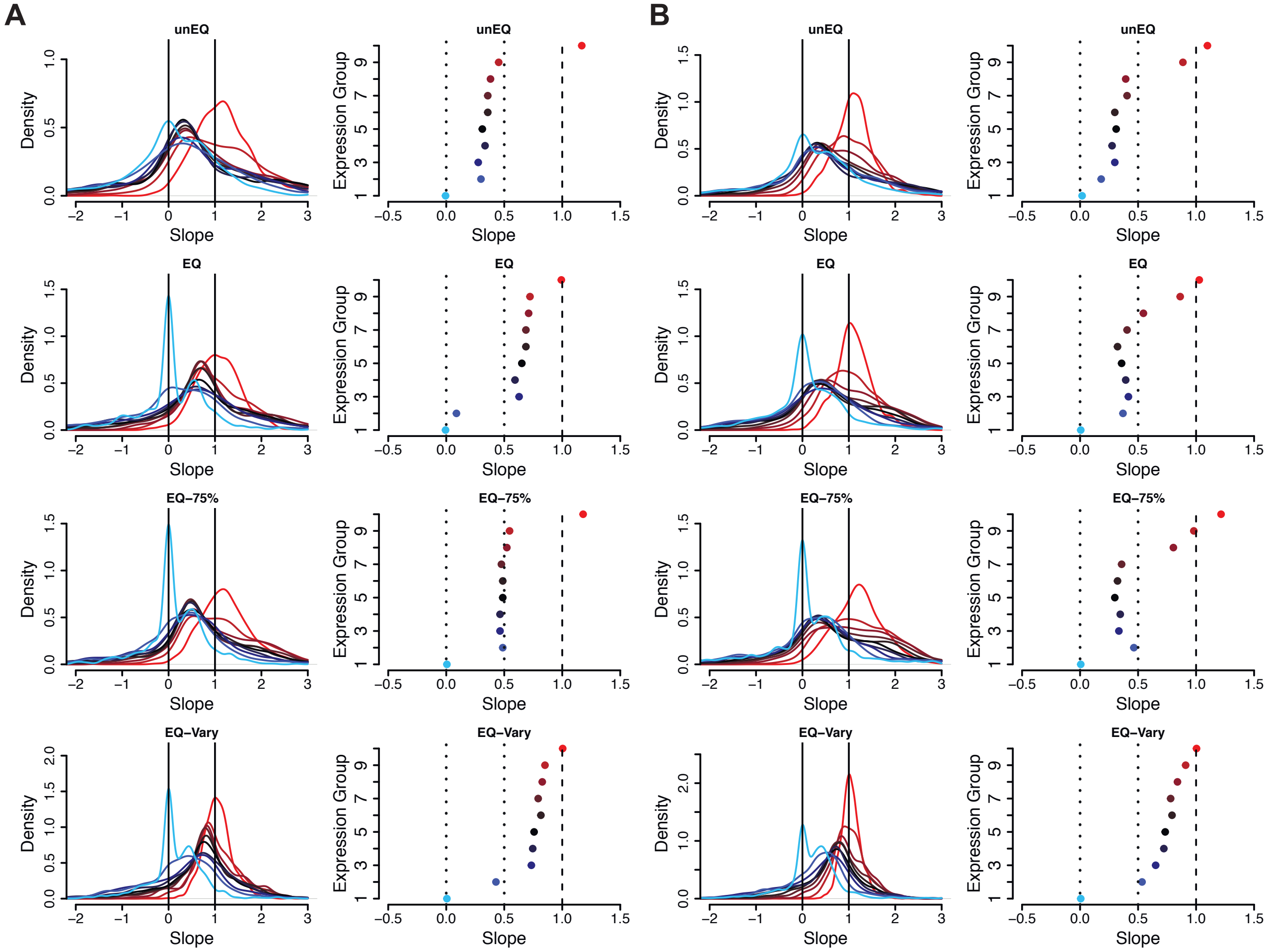

Supplementary Figure 9. Cell-specific and gene-specific properties are shown for datasets described in Figure 4 and the top portion of Table 1. The equalized datasets are Deng, Guo, and Picelli; the unequalized datasets are Shalek, Islam, and Chu.

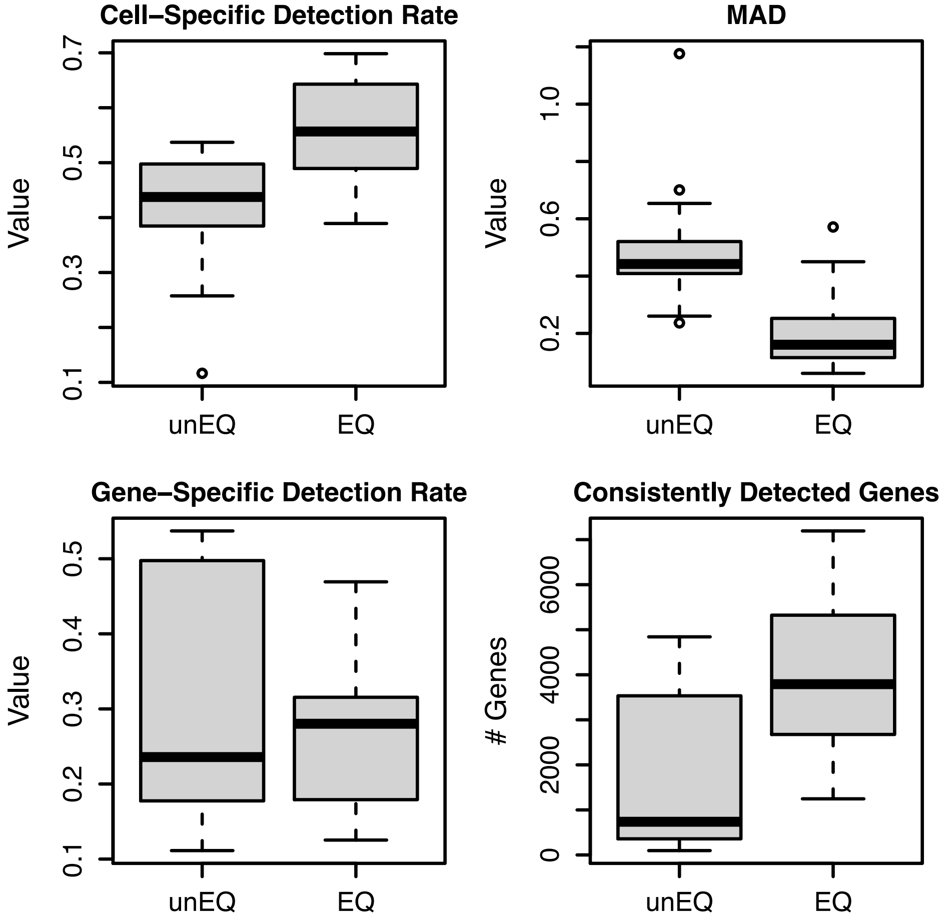

Supplementary Figure 10. Scaffold simulations of multiple cell type populations. A. Two populations with 50 cells each where 60% of genes were set to have distinct expression with fold-changes sampled from Normal(2, 0.4). B. Simulation of rare cell Population 2 with n=3 and a larger Population 1 with n=97; 80% of genes were set to have distinct expression with fold-changes sampled from Normal(2, 0.4). C. Simulation of three cell populations all with n=50. For Population 2, 60% of genes were set to have distinct expression with fold-changes sampled from Normal(2, 0.4). For Population 3, 40% of genes were set to have distinct expression with fold-changes sampled from Normal(1.5, 0.4).

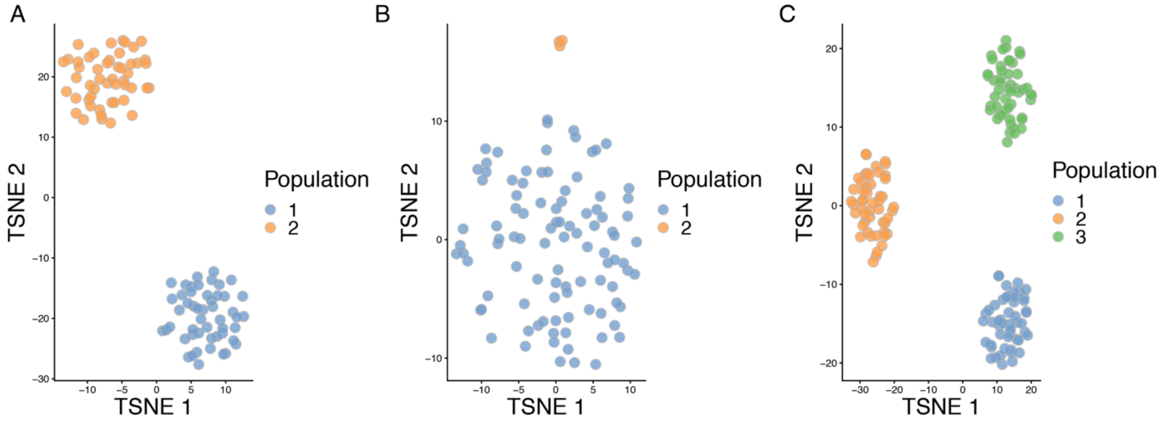

Supplementary Figure 11. Pairs of unequalized (unEQ) and equalized (EQ) experiments were simulated using Scaffold to have two cell populations (see Methods). The datasets were then embedded into two-dimensional spaces for visualization using either TSNE or EDGE and the silhouette distance was calculated. The silhouette distances were permuted for each simulated dataset to obtain a sampling distribution under the null hypothesis of no difference due to equalization. P-values (p) were calculated over 10,000 permutations. A. The permutation distribution of the proportion of equalized simulations having a larger silhouette distance on TSNE embeddings. B. The permutation distribution of the median silhouette differences between unEQ and EQ simulated data computed on TSNE embeddings. C & D. Similar to A & B using EDGE to embed the data into a two dimensional subspace.

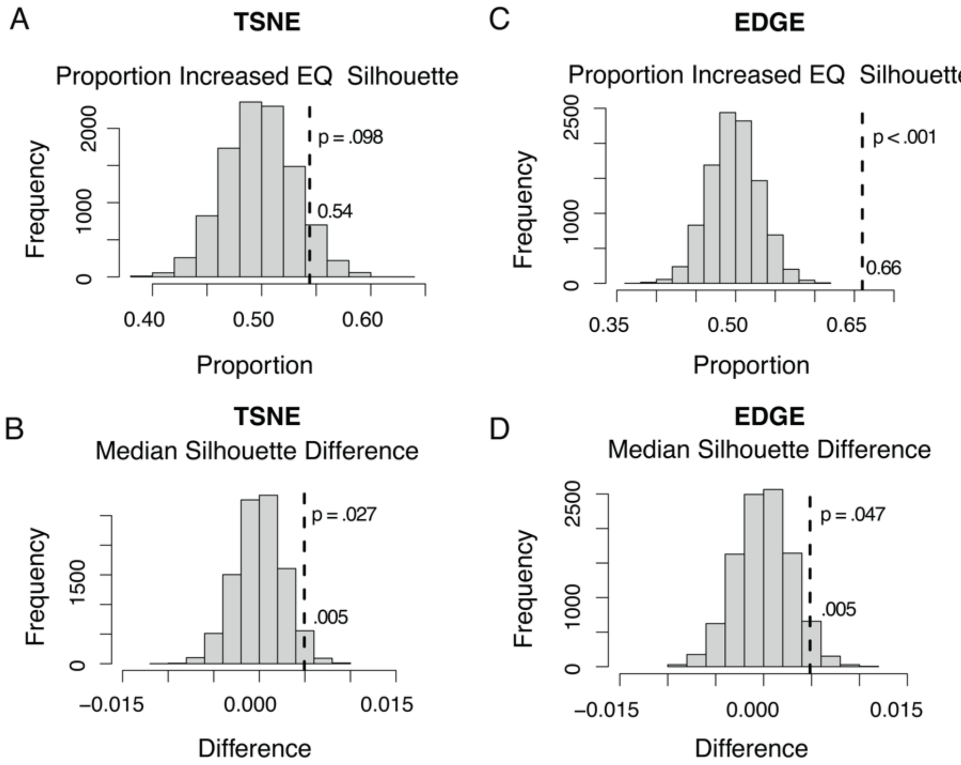

Supplementary Figure 12. Scaffold simulation of continuous population. A. Using the unEQ EC data as a reference, 100 cells were simulated without any dynamic genes. B. Scaffold simulated 20% of genes to have dynamic expression; no equalization was performed. C. Same simulation parameters as in B, but equalization was done.

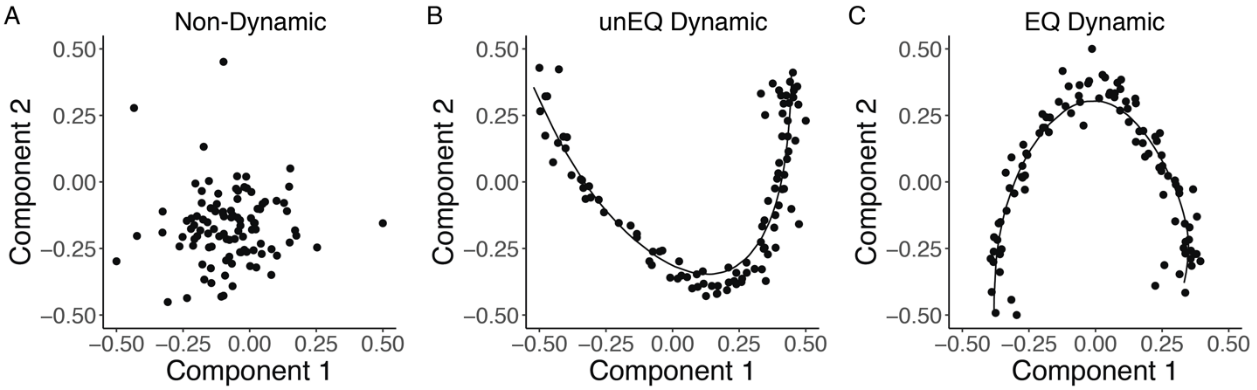

Supplementary Figure 13. A. Density plots of the distribution of estimated count-depth rates for the Grün-UMI dataset with genes grouped by expression level (left) and the mode of each group's slope distribution (right). The median absolute deviation (MAD) of the slope modes from one is used to quantify the variability in the count-depth rate. B-F. Cell-specific and gene-specific properties of the data simulated based on the Grün-UMI dataset. Gene-level properties are shown for a random sample of 200 genes.

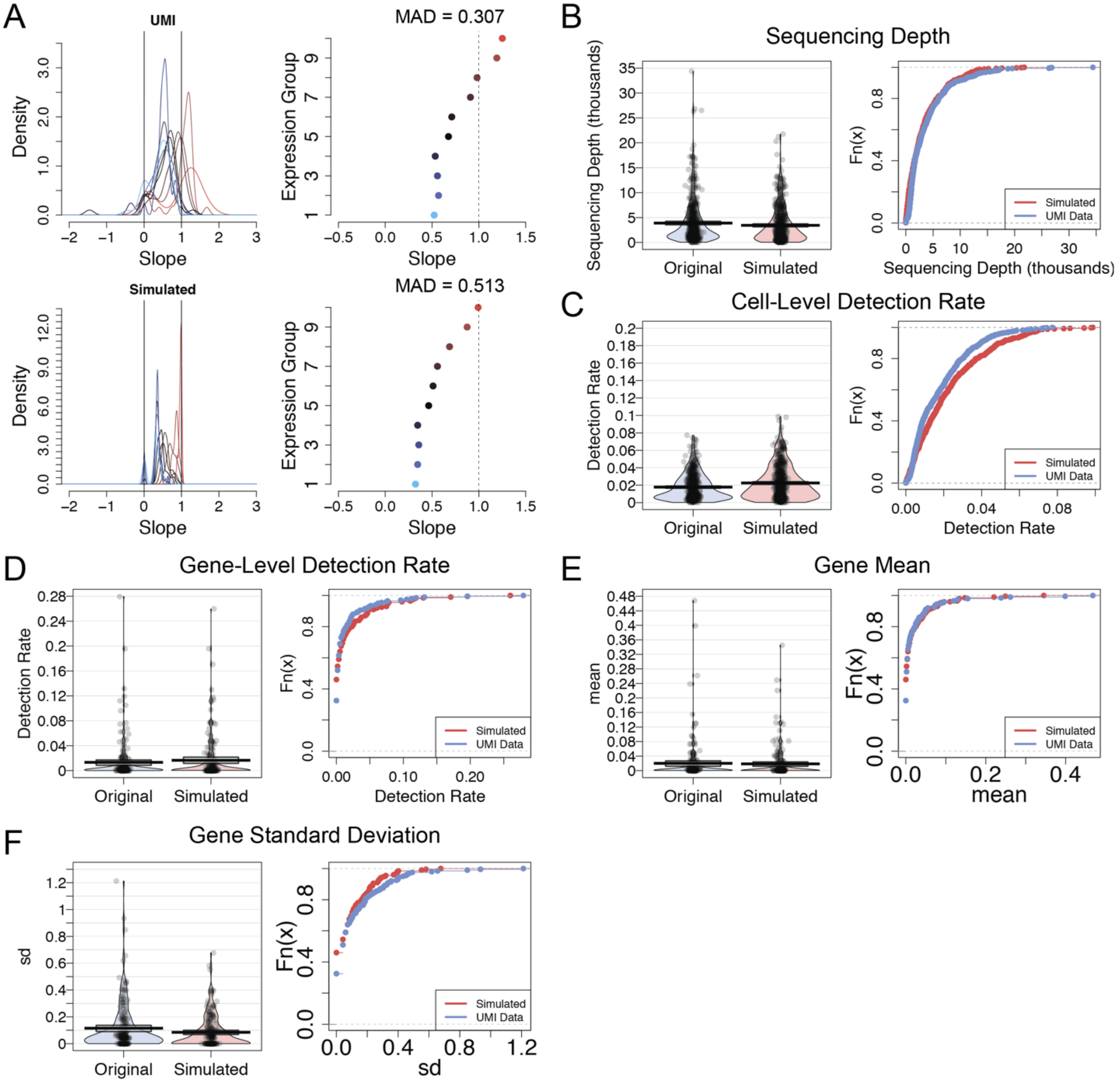

Supplementary Figure 14. A-E. Cell-specific and gene-specific properties of the data simulated based on the 10X dataset. Gene-level properties are shown for a random sample of 200 genes.

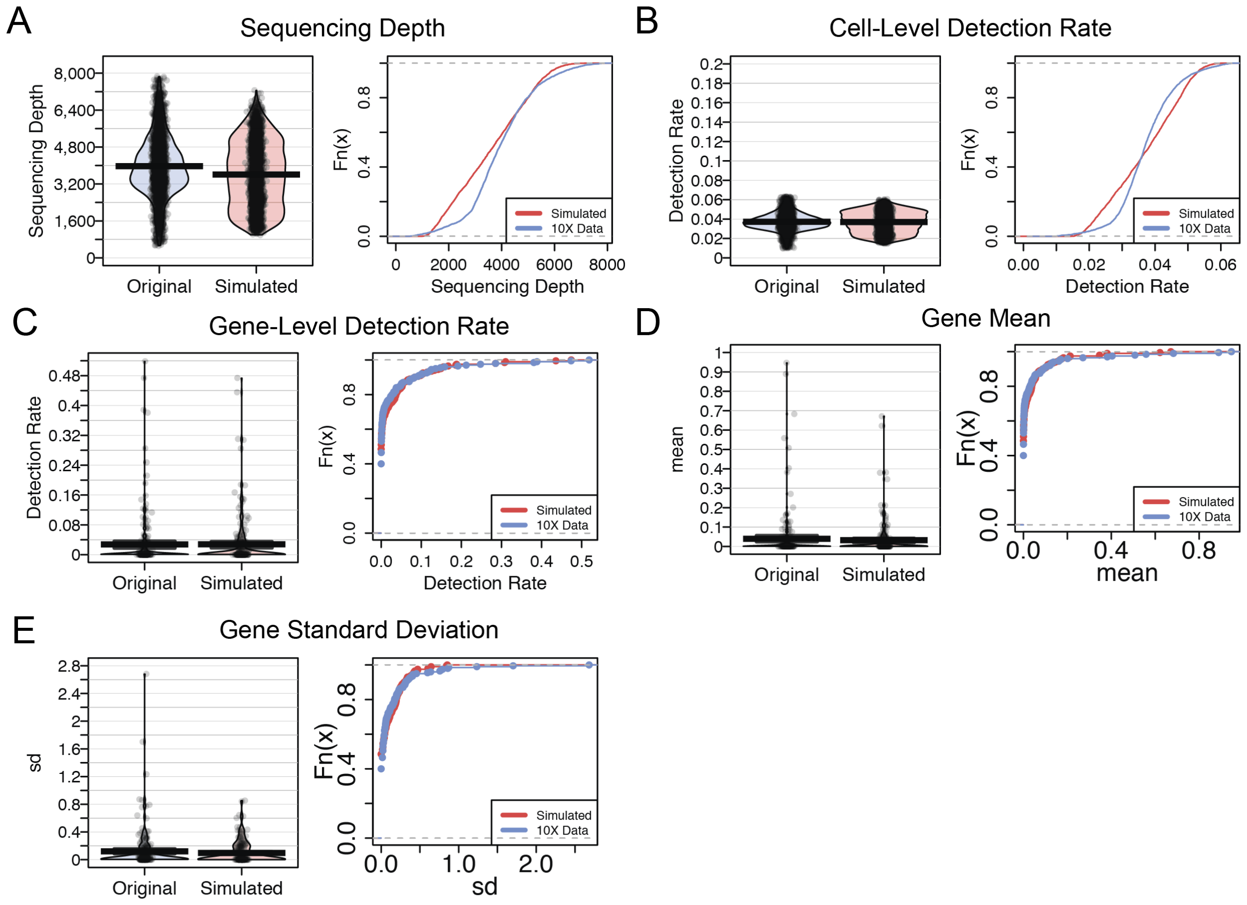

Supplementary Figure 15. A-E. Cell-specific and gene-specific properties of data simulated based on the Smart-seq3 HCA dataset. Gene-level properties are shown for a random sample of 200 genes.

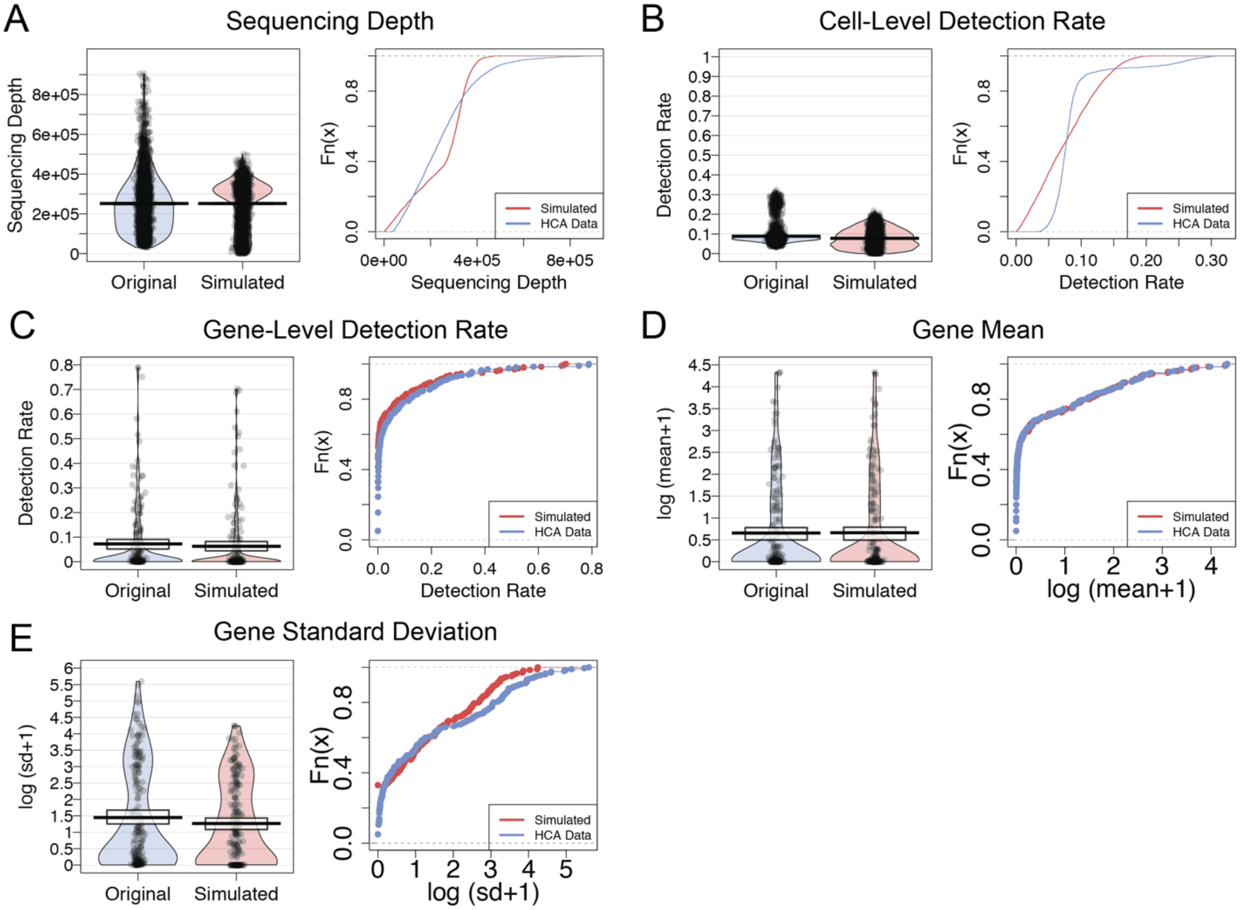

Supplementary Figure 16. Similar in structure to Supplementary Figure 14, but using the UMI counts from the Smart-seq3 HCA data and the UMI counts obtained in the simulated dataset.

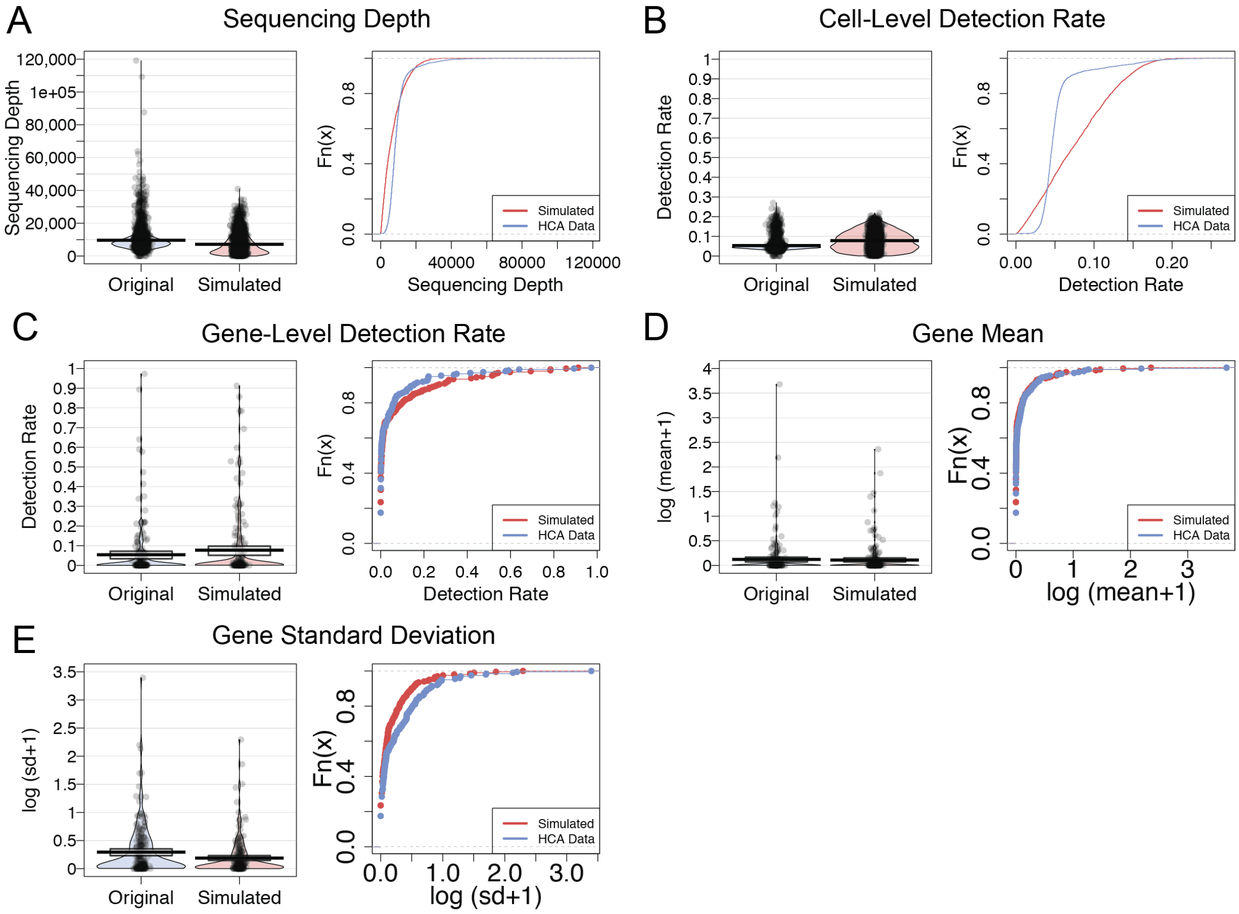

Supplementary Figure 17. A. Density plots of the distribution of estimated count-depth rates for the Smart-seq3 Fibroblast dataset with genes grouped by expression level (left) and the mode of each group's slope distribution (right). The median absolute deviation (MAD) of the slope modes from one is used to quantify the variability in the count-depth rate. B-F. Cell-specific and gene-specific properties of the data simulated based on the Smart-seq3 Fibroblast dataset. Gene-level properties are shown for a random sample of 200 genes.

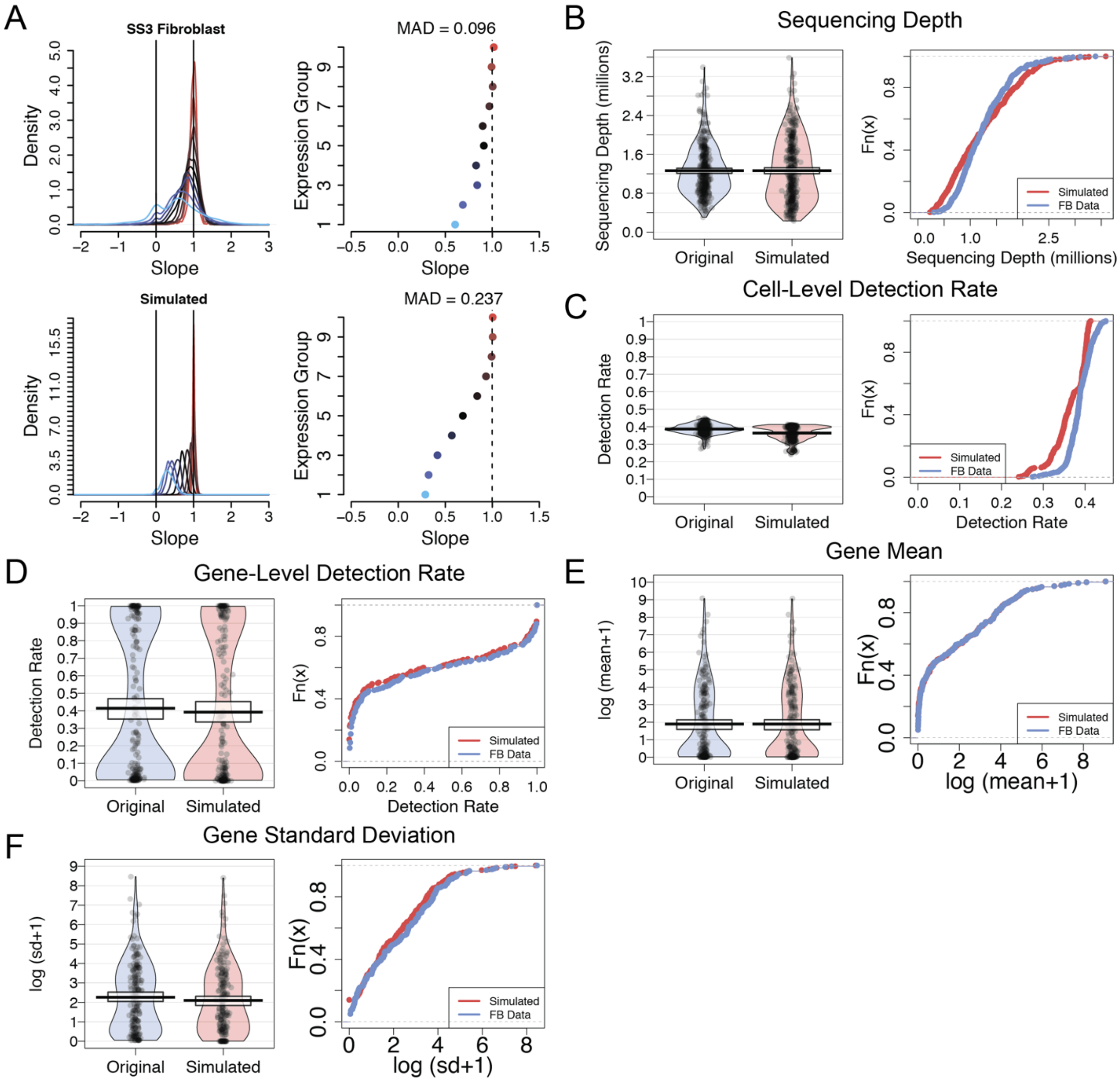

Supplementary Figure 18. Similar in structure to Supplementary Figure 16 using the UMI counts from the Smart-seq3 Fibroblast data and the UMI counts obtained in the simulated dataset.

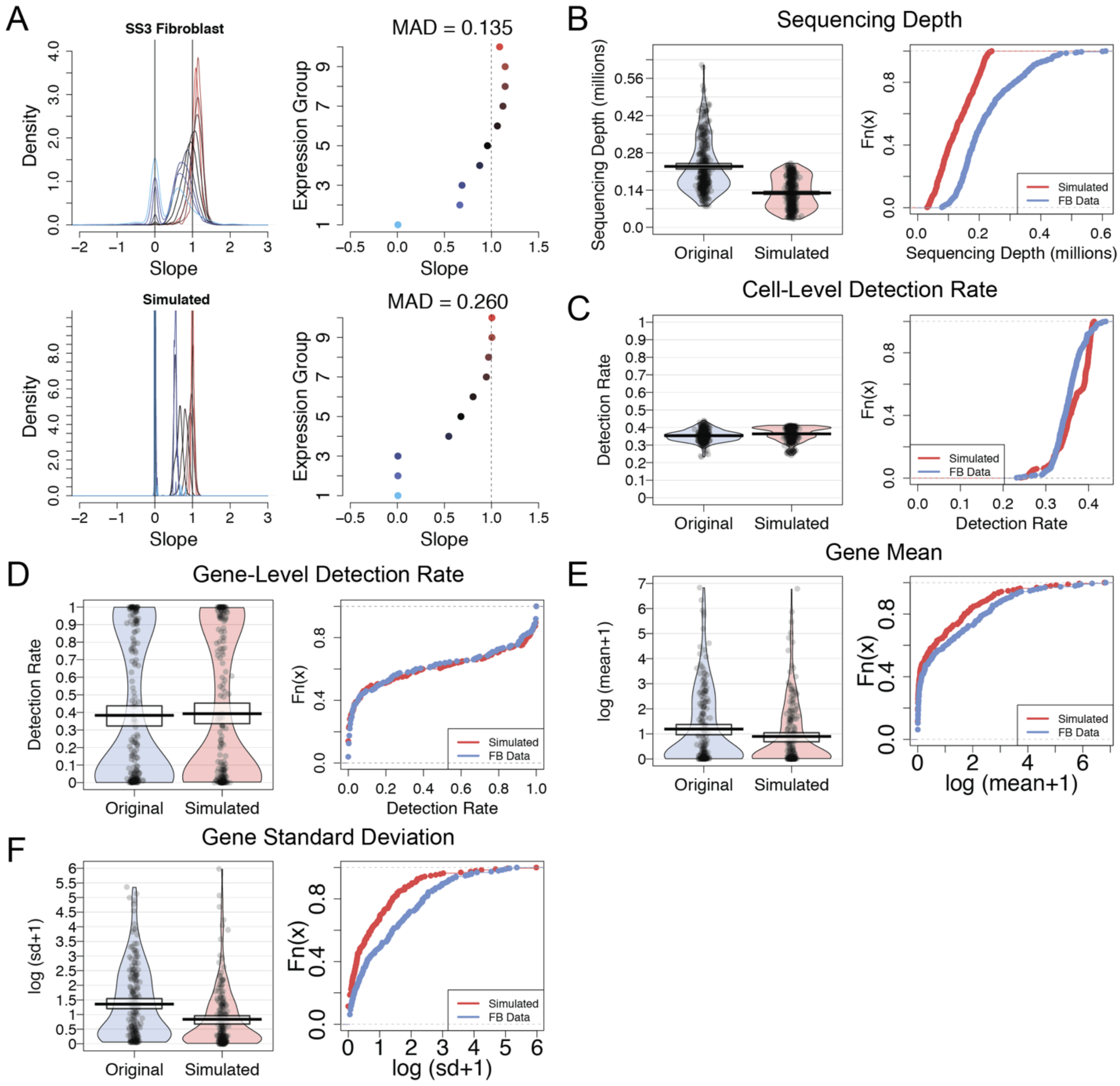

Supplement: gkab1071_Supplemental_Files [file gkab1071_supplemental_files.zip › SupplementaryMethodsAndFigures.pdf]
